# Maintenance of UK bread baking quality: Trends in wheat quality traits over 50 years of breeding and potential for future application of genomic‐assisted selection

**DOI:** 10.1002/tpg2.20326

**Published:** 2023-04-14

**Authors:** Nick S. Fradgley, Alison R. Bentley, Keith A. Gardner, Stéphanie M. Swarbreck, Matt Kerton

**Affiliations:** ^1^ Genetics and Pre‐Breeding Department National Institute of Agricultural Botany (NIAB) 93 Lawrence Weaver Road Cambridge UK; ^2^ International Maize and Wheat Improvement Center (CIMMYT) Carretera México‐Veracruz México; ^3^ DSVUK Top Dawkins Barn Banbury UK

## Abstract

Improved selection of wheat varieties with high end‐use quality contributes to sustainable food systems by ensuring productive crops are suitable for human consumption end‐uses. Here, we investigated the genetic control and genomic prediction of milling and baking quality traits in a panel of 379 historic and elite, high‐quality UK bread wheat (*Triticum eastivum* L.) varieties and breeding lines. Analysis of the panel showed that genetic diversity has not declined over recent decades of selective breeding while phenotypic analysis found a clear trend of increased loaf baking quality of modern milling wheats despite declining grain protein content. Genome‐wide association analysis identified 24 quantitative trait loci (QTL) across all quality traits, many of which had pleiotropic effects. Changes in the frequency of positive alleles of QTL over recent decades reflected trends in trait variation and reveal where progress has historically been made for improved baking quality traits. It also demonstrates opportunities for marker‐assisted selection for traits such as Hagberg falling number and specific weight that do not appear to have been improved by recent decades of phenotypic selection. We demonstrate that applying genomic prediction in a commercial wheat breeding program for expensive late‐stage loaf baking quality traits outperforms phenotypic selection based on early‐stage predictive quality traits. Finally, trait‐assisted genomic prediction combining both phenotypic and genomic selection enabled slightly higher prediction accuracy, but genomic prediction alone was the most cost‐effective selection strategy considering genotyping and phenotyping costs per sample.

## Core Ideas

- Baking quality of bread wheat varieties in the United Kingdom has increased despite declining grain protein content.
- Trends in frequencies of QTL alleles reflect trends in changes in wheat quality traits.
- Genomic selection proved a cost‐effective strategy for loaf baking quality traits compared to phenotypic prediction.

**Abbreviations**
1B/1R: Translocation of rye chromosome 1 to the short arm of wheat chromosome 1B
AACC: American Association of Cereal Chemists
AHDB: Agriculture and Horticulture Development Board
AIC: Akaike Information Criterion
BLAST: basic local alignment search tool
BLUP: Best Linear Unbiased Prediction
CIMMYT: International Maize and Wheat Improvement Center
DSV: Deutsche Saatveredelung
EG‐BLUP: Extended GBLUP
G×E: Genotype by environment interaction
GBLUP: genomic best linear unbiased estimate
GBM: Gradient boosted machines
GO: Gene ontology
GRIS: Genetic Resources Information System for Wheat and Triticale
HFN: Hagberg falling number
LASSO: Least Absolute Shrinkage and Selection Operator
Mb: Megabase
NABIM: National Association of British and Irish Millers
NIR: Near‐Infrared Reflectance
NMR: Nuclear magnetic resonance
PCoA: Principal coordinate analysis
Pina: Puroindoline a
Pinb: Puroindoline b
QTL: quantitative trait loci
RF: Random forest
RKHS: Reproducing kernel Hilbert space
SDS: Sodium dodecyl sulfate
SNP: single nucleotide polymorphism
TGW: thousand grain weight

## 1 INTRODUCTION

Bread wheat (*Triticum eastivum* L.) is a globally important food crop for human consumption, but is also used for livestock feed and biofuel (Shiferaw et al., 2013). Feeding grain crops that contain substantial human digestible protein to livestock is an inefficient use of increasingly scarce resources (Bentley et al., 2022). Enhancing the end‐use profile of wheat crops for food uses presents an opportunity to reduce the cost of feeding people per unit of land area and input use (Eisler et al., 2014; Wilkinson & Lee, 2018). High wheat yields are achieved in the United Kingdom (Calderini & Slafer, 1998) albeit with intensive fertilizer use (Lu & Tian, 2017), but only approximately one fifth of wheat varieties bred in the United Kingdom are high‐quality milling wheats for direct human consumption (AHDB, 2022), and the proportion of wheat grown in the United Kingdom for food has decreased to less than half in recent decades (Ray et al., 2022). Increasing pressures of climate change and demands of feeding a growing global population will necessitate increased production of high‐quality wheat varieties in highly productive northern growing regions such as the United Kingdom.

Since the early days of wheat breeding, a trade‐off between grain yield and grain quality has been observed (Percival, 1934), and there is strong evidence supporting a direct genetic trade‐off between grain yield and protein content (Michel et al., 2019; Scott et al., 2021; Simmonds, 1995). In wheat breeding programs with an explicit quality focus, simultaneous selection is carried out for a large number of milling and baking quality traits including protein content, high grain specific weight, high Hagberg falling number, good dough rheological properties such as high gluten strength and extensibility, large loaf baking size as well as good crumb structure, texture, and color. Testing the large number of important wheat milling and baking quality traits is expensive and time consuming, so these traits are generally tested in later stages of breeding programs once significant phenotypic and agronomic selection has already been applied. Some high‐throughput traits such as grain protein content and specific weight can be measured at earlier stages and are used to make selections and predict more expensive later stage traits. As these quality traits are often only imperfectly correlated to each other, forward selections to later generations are often unreliable and reduced genetic gain is achieved for any single‐trait. This challenge, in combination with ideal climatic conditions for obtaining high wheat yields, the wheat protein trade‐off, and availability of fertilizer (required to achieve high protein) have resulted in UK wheat breeders primarily focusing on breeding high yielding feed wheat varieties. Over the course of the last century, historical changes in agricultural practice, breeding approaches, and milling and baking processes have all evolved to adapt to this high yield focused wheat cultivation strategy (Belderok et al., 2000). Understanding the trends in past genetic gain in specific component quality traits can shed light on how crop improvements have historically been achieved and can also identify opportunities to inform future selection approaches for enhancing UK wheat quality.

Many breeding programs will run separate selection streams for feed and milling wheat end‐use classes. Here, selection of lines with high‐quality traits and/or high yield within crosses between milling and feed wheats are particularly important to achieve genetic gain in each stream. Higher throughput and low‐cost predictive traits, such as grain protein content and specific weight, are measured for the large number of lines at early‐stages in the breeding program and are used both to directly make early‐stage selections and to predict more costly later stage traits such as those obtained from loaf baking tests. Enhanced and earlier prediction and selection for these later stage traits would greatly improve capabilities for breeding high‐quality milling wheat varieties.

Developments in genotyping technologies have enabled advancements in genomics‐assisted breeding in wheat (Adamski et al., 2020). Characterization of specific genetic variants associated with traits through genome wide association studies have identified quantitative trait loci (QTL) that can be used for marker‐assisted selection in breeding programs. The role of the three homoeologous high‐molecular‐weight glutenin genes in determining wheat quality was identified by Payne et al. (1982), and genetic markers for these loci are now routinely used in breeding programs (Liang et al., 2010; Lupton, 2005). Likewise, identification of the genetic control of endosperm texture and grain hardness by the *Puroindoline a (Pina)* and *Puroindoline b (Pinb)* loci (Morris, 2002) and of the negative effects of the 1B/1R translocation from rye on Hagberg falling number and gluten strength (Graybosch, 2001) have proved to be highly applicable in wheat breeding. More recently, genetic control of quality traits for human health, such as increased dietary fiber (Ibba et al., 2021), have been identified and can be employed in breeding. These examples highlight the usefulness of QTL that explain a large proportion of the heritable trait variation and for traits with relatively simple genetic architectures. However, genetic control of the majority of more complex and highly polygenic quality traits such as grain protein content and flour water absorption have been much more difficult to explain by single QTL genetic effects (Fradgley et al., 2022; Kristensen et al., 2018; Scott et al., 2021). For such traits, genomic selection (Meuwissen et al., 2001) is the preferred approach and has enabled great progress in accelerating genetic gain in modern crop breeding programs (Crossa et al., 2017; Voss‐Fels et al., 2019). A wide diversity of genomic prediction models have been developed (Howard et al., 2022) so that testing and selection of the best model for specific trait architectures and breeding scenarios as well as design of the training panel are required for optimum implementation of genomic selection. The costs and benefits of implementing genomic selection must also be pragmatically assessed with regard to the logistics of current breeding pipelines. This is particularly true for making accurate comparisons between phenotypic selection using high throughput phenotyping methods (Robert et al., 2022) and genotyping costs for the number of breeding lines tested at different stages of a commercial breeding program. Genomic selection should be able to increase the accuracy of early‐stage selection enough to allow faster recycling of material for crossing to initiate new breeding cycles, and hence accelerate genetic gain.

Here, we assembled a panel of historic high‐quality UK wheat varieties combined with breeding lines from multiple recent cycles from the Deutsche Saatveredelung (DSV) UK breeding program to examine the potential application of novel QTL for marker‐assisted selection. We also used the panel to test genomic selection to enhance genetic gain for high milling and baking quality varieties within the context of a commercial breeding program. To evaluate how progress in UK quality wheat breeding has historically been made and how selection could be enhanced in the future, we (i) investigated temporal trends in milling and baking quality traits among genotypes in the panel released over the period 1935 to 2020, alongside trends in marker‐based kinship among historical cohorts of high‐quality wheat lines; (ii) performed a genome wide association study to identify potentially new QTL associated with quality trait variation; (iii) examined trends in frequency of positive alleles of QTL identified over recent decades of breeding and analyzed examples of selection in the historic wheat pedigree to determine how identified QTL have been, or could be, exploited for marker‐assisted selection in breeding; and (iv) tested the prediction accuracy of a diverse set of genomic selection models and compared them to the current approaches of phenotypic prediction of final loaf baking and milling quality traits based on early‐stage predictive quality traits and trait‐assisted genomic prediction that combined both genomic and phenotypic data.

**Core Ideas**

- Baking quality of bread wheat varieties in the United Kingdom has increased despite declining grain protein content.
- Trends in frequencies of QTL alleles reflect trends in changes in wheat quality traits.
- Genomic selection proved a cost‐effective strategy for loaf baking quality traits compared to phenotypic prediction.

## 2 MATERIALS AND METHODS

### 2.1 Germplasm

A panel of 379 wheat genotypes was assembled to represent genetic diversity in high‐quality commercial wheat varieties over recent decades and within a present‐day UK and German winter wheat breeding program. These consisted of five groups: (i) 28 previously released commercial wheat varieties that have appeared as either National Association of British and Irish Millers (NABIM) group 1 or 2 milling quality varieties for bread making on the Agriculture and Horticulture Development Board (AHDB) recommended lists between 2004 and 2019 (https://ahdb.org.uk/knowledge‐library/recommended‐lists‐archive); (ii) 45 older and historical released spring and winter wheat varieties that were part of the already available wheat diversity panel as outlined by Fradgley et al. (2019) and noted as high quality by either Belderok et al. (2000) or listed as “Excellent,” “High,” or “Good” quality on the Genetic Resources Information System for Wheat and Triticale (GRIS; http://wheatpedigree.net/); (iii) 10 additional high‐ and low‐quality advanced breeding lines and released varieties that have recently been used for crossing within the DSV UK breeding program; (iv) 176 advanced breeding lines from six breeding cycles of the DSV UK breeding program; and (v) 131 advanced breeding lines from two cycles of the DSV breeding program in Germany. The names of the recent and historical released varieties used in the panel are given in Table S1.

### 2.2 Field trials

Reflecting standard breeding assessment capacity and timeframes, two main field trials were conducted over two growing seasons (2019–2020 and 2020–2021; hereafter denoted year 1 and year 2), at the DSV UK breeding station in Wardington, Oxfordshire, UK (Latitude = 52.103326; Longitude = −1.2827740). Plots were managed according to field standard practice including fungicides to control foliar diseases, herbicides to control weeds, and a total of 200 kg ha^−1^ of nitrogen fertilizer applied over three applications (50, 100, and 50 kg N ha^−1^, respectively). A total of 223 genotypes were tested in both years, with an additional 21 genotypes tested in year 1 only (244 genotypes in total tested in year 1) and an additional 28 genotypes tested in year 2 only (251 genotypes in total tested in year 2). Two replicate plots were planted for 56 genotypes (year 1) and 49 genotypes (year 2) while the remaining genotypes were arranged in single unreplicated plots. Both trial years consisted of 300 small plots (1 m^2^) in nested sub‐blocks design where in year 1, there were 10 sub‐blocks of 10 plots nested within three main blocks of 100 plots each. In year 2, there were two sub‐blocks of 10 plots nested within each of 15 main blocks of 20 plots each. Trial design was established to closely replicate current testing conditions in a commercial breeding context. Trial plots were harvested with a plot combine, and grain samples were cleaned with an aspirator cleaner and used for further quality analysis.

In addition, data from quality assessments on advanced breeding lines from five trial years from 2017 to 2021 in the DSV UK breeding program were included. These included from 13 to 119 genotypes per year, of which two or three were control varieties common across all trials. These breeding program trials were also conducted at the DSV UK breeding station at Wardington. Across all trial datasets (both main trials and 5 years of breeding program trials), a total of 379 wheat genotypes were a median of seven, and minimum of two genotypes were in common between trials so that analyses across environments could be made.

### 2.3 Phenotyping and grain quality assessment

Grain samples from all trial plots were assessed for a range of traits that are predictive of specific aspects of milling and baking quality in line with methods approved by the American Association of Cereal Chemists (AACC, 2000). These included grain protein content measured using near‐infrared reflectance (NIR) (AACC method 39‐25.01), as well as specific weight (AACC method 55‐10.01), both measured on grain using a Foss, Infratec™ NOVA. Alpha amylase activity was assessed using the Hagberg falling number (HFN) test (AACC approved method 56–81.03). Grain morphology traits including mean grain length, width, area, and estimated thousand grain weight (TGW) were assessed on 300 to 400 grains per sample using a MARVIN seed analyzer (GTA Sensorik GmbH).

A subsample of 108 genotypes in year 1 and 96 genotypes in year 2 (15 in common between years), as well as 20 additional advanced breeding lines from other DSV UK breeding program trials, giving a total of 209 genotypes, were assessed for milling and loaf baking quality. As is common within the DSV UK breeding program, samples from each trial were taken from single field plots with no replicated genotypes within each year to maximize the number of tested genotypes for low throughput and expensive phenotyping and within field environmental effects were assumed to be negligible. Grain was milled using a Laboratory Buhler roller mill (AACC approved method 26‐21.02), and extraction rate was determined as the percentage of standardized weight of white flour fraction yielded from the total weight of grain milled. Dough rheology traits were then assessed with the milled white flour using a Calibre, DoughLab 2500 following the AACC approved method 54–70.01 where 50 g of flour (adjusted for moisture content) was mixed under high speed for 10 min. The traits assessed by this method include adjusted water absorption required to reach a peak mixing torque of 6.5 N m, dough development time, stability, and softening.

**FIGURE 1:**
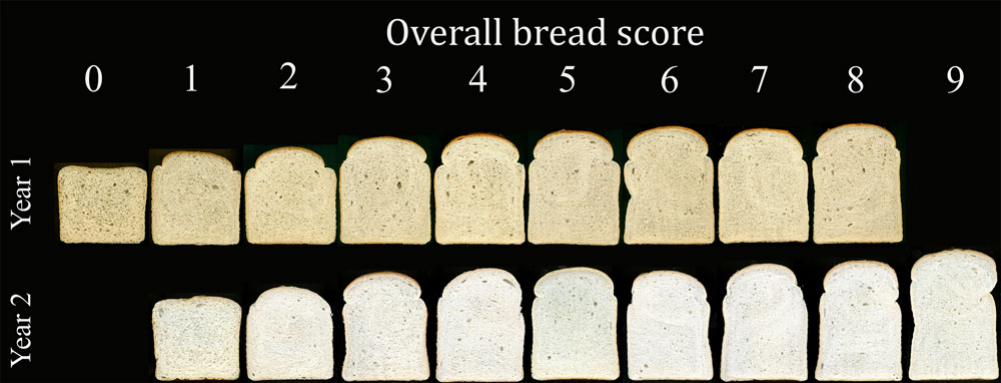
Examples of loaves baked with the Chorley Wood Baking Process for year 1 and year 2 experiments. Numbers indicate the overall bread score given to each loaf.

Test loaves were then baked with the refined white flour in a highly controlled, industry standard testing lab using a small‐scale version of the Chorley Wood Baking Process (Cauvain & Young, 2006). This baking method was used as it is the standard method used to assess wheat baking quality in the United Kingdom. The method involved baking two test loaves for each sample with 600 g of flour. Expected water absorption was determined by near infrared reflectance (NIR) analysis of flour samples. Other ingredients were included as (w/w) percentages of the flour weight: fluid shortening (1%), bakers’ yeast (2.7%), sodium chloride salt (1.55%), dough improver containing protease and amylase enzymes to boost yeast activity and ease dough expansion during proofing, oxidizing agents and emulsifiers (2%), soya flour (1.5%), and calcium carbonate (0.22%). Dough samples were mixed in a high‐speed rotary mixer with a work input of 15 Wh/kg, while controlling the temperature of the dough at 28 °C (± 0.5°C) with a water cooler. Dough sub‐samples of 894 g were then molded, placed in baking tins, and proved for 55 min at 43°C and 75% humidity. Loaves were then baked at 235°C for 25 min in a deck oven. Loaf bake height was recorded immediately after baking, and the oven spring was calculated as the difference between bake height and the height of the dough after proving. After leaving baked loaves to cool for 2 h or until the internal temperature reaches 28°C, loaf volume was measured by three‐dimensional laser topography scanning using AACC Method 10–14.01. The color of the sliced crumb was quantified by tristimulus color measurement using a Minolta CR310 including the L* (white vs dark) and b* (yellow vs blue) color space axis as well as calculating L*‐b* as a derived value of overall color quality. Visual assessments of loaf quality characteristics were also made independently by two professional bakers at the test lab of Allied Bakeries, Maidenhead, UK. Crumb structure was scored on a scale of 0 to 5 from “weak,” “firm,” or “doughy” to “resilient.” Crumb texture was scored on a scale of 1 to 4 from “coarse” to “fine.” A final overall bread quality score integrating all other loaf quality assessments was also given on a scale of 0 to 9 (Figure 1).

### 2.4 Trials analysis

Best linear unbiased prediction (BLUP) predicted means were calculated for each genotype for each baking quality trait across all trials and within each of the two main trial years. BLUPs were calculated by fitting mixed models using the “lme4” package in R. For analyses within each main trial year, effects of blocking structure could be accounted for with Equation (1):

(1) $$Y_{ik}=\;\mu +G_{i}+Bb_{k}+e_{i},$$

where *Y* is a vector of observed values in the response trait, μ is the overall mean, *G* is the random effect of the *i*th genotype, Bb is the random effect of the *k*th sub‐block nested with main block, and *e* is the residual error. For the meta‐analysis across all trials, Equation (2) was used for predictive quality traits that had some replication within each environment:

(2) $$Y_{ij}=\;\mu +G_{i}+E_{j}+GE_{ij}+e_{ij},$$

where, in addition to the components of Equation (1), *E* is the random effect of the *j*th trial environment, and GE is the random genotype by environment interaction effect for the *i*th genotype and the *j*th trial environment. Blocking structure was not included in unreplicated breeding program trials so blocking effects could not be included in these analyses across all trials. For milling and baking test traits that were not replicated within each environment, a similar model was fitted but without the genotype by environment interaction effect.

Variance components were extracted from fitted mixed models for each trait, and the broad sense heritability (*H* ^2^) for each trait across all trials was estimated using Equation (3):

(3) $$H^{2}=\frac{{\sigma^{2}}_{G}}{{\sigma^{2}}_{G}\;+\;\frac{{\sigma^{2}}_{GE}}{nE}\;+\;\frac{{\sigma^{2}}_{e}}{np}}\;$$

where ${\sigma^{2}}_{G}$ is the genetic variance, ${\sigma^{2}}_{GE}$ is the genotype by environment variance, nE is the number of trial environments, ${\sigma^{2}}_{e}$ is the residual error variance, and np is the mean number of plots per genotype.

### 2.5 Genetic markers

The panel was genotyped with a custom 25k single nucleotide polymorphism (SNP) array (provided by SGS—Institut Fresenius TraitGenetics) that was a combined subset of 14,455 markers from the Illumina 90k wheat SNP array (Wang et al., 2014), 2,949 markers from the Affymetrix Axiom 35k wheat breeders’ array (Allen et al., 2017), 5,536 markers from another novel and unpublished 135k Affymetrix SNP array, as well as 265 trait specific markers from the literature. From the full set of markers, a subset of 18,493 were selected that had less than 10% missing data and minor allele frequency over 0.05. Remaining missing data were imputed using random forest with the “missForrest” package (Stekhoven & Bühlmann, 2012).

Physical positions on the Chinese Spring RefSeq v1.0 reference genome (IWGSC et al., 2018) for each marker were determined by a basic local alignment search tool (BLAST; Altschul et al., 1990). Correct chromosome assignments were confirmed by comparing linkage disequilibrium values of a marker against all other markers across all genotypes in the panel. For each marker, a set of linked markers (*R* ^2^ > 0.5) was found and the most common consensus chromosome assigned. If this consensus chromosome was different to that of the focal marker assigned chromosome, then it was corrected to the best BLAST hit on the linked markers consensus chromosome.

### 2.6 Wheat panel genetic analysis and diversity trends

A linear kinship matrix for the panel of lines was calculated using the A.mat function in the rrBLUP R package (Endelman, 2011) for a subset of 4,970 SNP markers, that were the result of pruning the full set of markers by removing one of each pair of markers that correlated at *R* ^2^ > 0.8. Principal coordinate analysis (PCoA) was then conducted on this relationship matrix, and the genotype weightings on the first two dimensions were plotted to visualize relationships among genotypes and groups of lines.

Trends in genetic diversity among released wheat varieties were evaluated between the release years 1935 and 2020. For this, the mean and median kinship coefficients were calculated for each year for genotypes with a year of release within a sliding window of 20 preceding years. Mean and median kinship coefficients were also calculated within and across each breeding cycle of DSV UK and German breeding lines.

### 2.7 Genome‐wide association mapping

Quantitative trait locus (QTL) mapping was performed by genome‐wide association for each trait BLUP using the full set of 18,493 markers with the GWASpoly package (Rosyara et al., 2016) in R analysis software. Meta‐analysis was performed for trait BLUPs across all trials as well as individually for each of the two main trials. The covariance matrix for polygenic effects was estimated using “the leave one chromosome out” method, and population structure was accounted for by including the first three principal components of the marker data as covariates. Thresholds for defining significant marker trait associations at the 90% confidence interval were estimated from 500 permutations of randomly sampling data for each trait. QTL intervals were defined for each QTL identified for each trait as the closest left and right flanking markers that are outside of those within the QTL interval (defined as on the same chromosome, in linkage disequilibrium with *R* ^2^ > 0.5 to the peak marker and with either a −log10(*p*) value within 2 −log10(*p*) of the peak marker or above the permutation‐based significance threshold for that trait). Similarly to Fradgley et al. (2022), all significant marker trait associations above the threshold were grouped, where pairs of peak markers that had squared correlation coefficients (*r* ^2^) above 0.5 were considered as collocating QTL across all traits. Hierarchical clustering using the complete linkage method was then performed using a pairwise distance matrix where pairs of markers that were considered the same QTL had a value of 0 while pairs that were not had a value of 1. All associated markers were then assigned to QTL groups using the cutree function in R using 0.5 as the height threshold. For each trait, all QTL peak markers with the greatest significance per QTL group were fitted in a full multi‐QTL model using the fit.QTL function in GWASpoly to test QTL effects by likelihood ratio tests under backward elimination and to determine partial R^2^ effects and fitted −log10(*p*) values for each QTL peak marker. All fitted QTL peaks with a −log10(*p*) value below 3 were removed.

Gene content between left and right QTL flanking markers physical map positions was extracted using the Ensemble Biomart resource (Kinsella et al., 2011) for the IWGS Chinese Spring RefSeq v1.0 genome (IWGSC et al., 2018) for all QTL with intervals less than 10 Mb. Intervals were averaged for pleiotropic QTL found for multiple traits. Locations of likely candidate genes already described in the literature was compared using gene sequences available on the National Center for Biotechnology Information nucleotide database and Basic Local Alignment Search Tool (BLAST; Mount, 2007) search comparisons to the wheat RefSeq v1.0 genome (IWGSC et al., 2018).

### 2.8 QTL historic selection trends

Trends over time in marker allele frequencies of QTL peak markers in released milling wheat varieties were examined to determine putative historical selection by breeders. Allele frequencies of the positive allele at each consensus QTL were calculated for released varieties with a 20 preceding year sliding window between 1970 and 2020. The trend was quantified by linear regression of the changes in allele frequency over time.

Specific selection effects by breeders over the historic pedigree were also examined by comparing QTL allele states of the released varieties with that of their parents. Pedigree data were used from the latest version of NIAB wheat pedigree resource (Fradgley 2021) as outlined by (Fradgley et al., 2019). For each QTL, a subset pedigree of simplex families was found where the QTL allele status for released milling quality varieties was different to one of its two parents. Even when marker information was only available for one of the parents, it can be deduced that the released variety was selected by a breeder from a family of segregating lines with a 50% probability of inheriting either QTL allele between polymorphic parents assuming Mendelian inheritance. To increase the number of simplex families that could be considered, parental genotypes were inferred if grandparent genotypes were known to be monomorphic in the pedigree.

### 2.9 Genomic prediction models

A diverse set of genomic prediction models that represent contrasting approaches to genomic prediction and modeling various genetic architectures were tested for each quality trait measured across all trials. Cross validation was performed to determine prediction accuracy with five rounds of 10‐fold random validation assignment of genotypes where for each fold, models were trained with data from a random sample of 90% of the lines and predictions tested for the remaining 10% of data. In addition, cross validation was performed using breeding lines from each cycle of the DSV UK breeding program as test sets with models trained on all remaining data. Prediction accuracy was estimated as the Pearson's correlation coefficient (r) between the observed BLUP data and the predicted trait values across all cross‐validation folds for each of the five rounds of random cross validation and for each breeding program cycle. The subset of 4970 pruned SNP markers were used for all prediction models. For fair comparison, predictions were made within the same cross validation assignments for all models tested. Six prediction models were tested as follows:

(i) The baseline genomic best linear unbiased estimate (GBLUP) prediction models were implemented using the rrblup package (Endelman, 2011) in R. This includes a linear additive genomic relationship matrix (*G*) defined using the A.mat function as described by (VanRaden, 2008) and Equation (4):

(4) $$G\;=\frac{MM^{\prime }}{p},$$

where *M* is the n × m marker matrix and *p* is the number of markers.

(ii) An extension of the GBLUP (EG‐BLUP) was implemented that aims to integrate epistatic marker effects (Jiang & Reif, 2015) with an additional relationship matrix (*H*) that is the Hadamard product of *G* with itself: H=G⊙G (⊙ indicates the Hadamard product). EG‐BLUP was implemented with Bayesian regression models in the BGLR package (Pérez & Campos, 2014) in R. Both *G* and *H* matrices were fitted as kernels with 5000 iterations of the Gibbs sampler of which 1000 were burn‐in and a default thinning value of five.

(iii) Reproducing kernel Hilbert space (RKHS) regression models were also fitted which model the genomic relationship matrix using non‐linear Gaussian kernels. The Gaussian kernel (*K*) was defined as the exponential relationship between the Euclidian distance among genotypes with Equation (5)

(5) $$K\;=\;\exp\left(-hD_{ii^{\prime }}^{2}/Q\right),$$

where $D_{ii^{\prime }}^{2}$ is the squared Euclidean distance of the n × m marker matrix, *Q* is the mean distance, and *h* is the bandwidth parameter as outlined by (Costa‐Neto & Fritsche‐Neto, 2021; Montesinos‐López et al., 2021). The kernel averaging approach was taken by fitting multiple Gaussian kernels with different *h* values where h=1/m×{1/5,1,5} where *m* = the median of the squared Euclidean distance (de los Campos et al., 2010; Pérez & Campos, 2014). The same parameters of the Gibbs sampler were used as for EG‐BLUP.

(iv) Least absolute shrinkage and selection operator (LASSO) models were used which assign linear additive effects to all SNP markers, but most SNP effects are shrunken to zero. Models were fitted within the glmnet package (Friedman et al., 2010) in R. Optimum values of lambda were determined for each model by eightfold cross validation of each training dataset for each model for 50 lambda values between 1 × 10^−24^ and 0.4096.

The following non‐parametric ensemble and machine learning models were also used:

(v) A fast implementation of random forest (RF) models was fitted in the ranger package (Wright & Ziegler, 2017) in R. For each RF model, 500 regression trees were grown with unlimited interaction depth, but with a minimum leaf node size of 15, and one third of the predictor SNP variables were randomly sampled to be considered for each tree split.

(vi) Stochastic gradient boosted machines (GBM) models (Friedman, 2002) were implemented in the gbm package (Ridgeway, 2007) in R. Performance of these models vary greatly with different hyperparameters, so a forward prediction tuning step was used to determine the optimum hyperparameters to use for fitting each model. This involved setting up a large grid search space including tree interaction depth values of 2, 3, 4, 5, and 6; the bagging fraction of the training set randomly sampled at each step of 0.4, 0.5, 0.6, 0.7, and 0.8; and a sequence of five minimum leaf node size values between 2 and one twentieth of the number of individuals in the training set. Validation error was initially determined for a random sample of 15 components of the grid search space for GBM models with a single random 80:20 split of the training and validation factions. These models for hyperparameter optimization were run with only 50 iterations of regression trees and a large shrinkage value of 0.2 to minimize computation time in this tuning phase. The 15 values of validation error and the sets of three hyperparameters used to fit those models were then used as response and explanatory variables, respectively, in random forest models for forward prediction of the next best set of grid search hyperparameters to try with the lowest predicted validation error. This forward prediction and hyperparameter search iteration was repeated five times to ensure efficient and fast searching and testing of the large hyperparameter space. Once optimum estimated values of interaction depth, bagging fraction, and minimum leaf node size were determined, these parameters were used for larger and slower GBM models with 500 tree iterations and a lower shrinkage value of 0.03. The optimum tree iteration from which to make final predictions was determined by fivefold cross validation to minimize overfitting.

### 2.10 Comparing phenotypic and trait‐assisted genomic prediction

Current practice within the DSV UK breeding program uses phenotypic data for predictive quality traits measured on large numbers of lines at early generations to predict and make selections for overall baking quality measured at later stages. Therefore, the potential to predict later stage milling and baking quality traits was assessed using either first‐stage quality traits (including grain protein content, specific weight, and HFN) or second‐stage quality traits (additionally including dough development time, stability and softening) in comparison to single‐ or multi‐trait genomic prediction models as well as trait‐assisted genomic prediction, which combines both genomic data and early‐stage phenotypes.

Multiple linear regression was used for phenotypic prediction models where predictive quality traits from either first and/or second stages were fit as explanatory variables with each later stage milling and baking quality trait as response variables. Stepwise model simplification was implemented using the step function in R to remove non‐significant (*p* > 0.05) explanatory variables and to select a fitted model with the lowest Akaike information criterion (AIC). These phenotypic predictions were compared with single‐ and multi‐trait Bayesian linear regression genomic prediction models that were fitted with the BGLR package in R (Pérez & Campos, 2014) using a linear genomic relationship matrix as outlined above. Separate models for single‐trait models while multi‐trait models were fitted using the Multitrait function, as described by Pérez‐Rodríguez and Campos (2022), where all trait data for test genotypes were masked as missing. In addition, we tested trait‐assisted genomic prediction models; all trait data except the predictive quality traits for either first or second stage groups were masked for test genotypes. Models used 10,000 iterations of the Gibbs sampler, of which 1000 were burn‐in.

## 3 RESULTS

### 3.1 Genetic structure reflects spatial and temporal trends in breeding for end‐use quality

**FIGURE 2:**
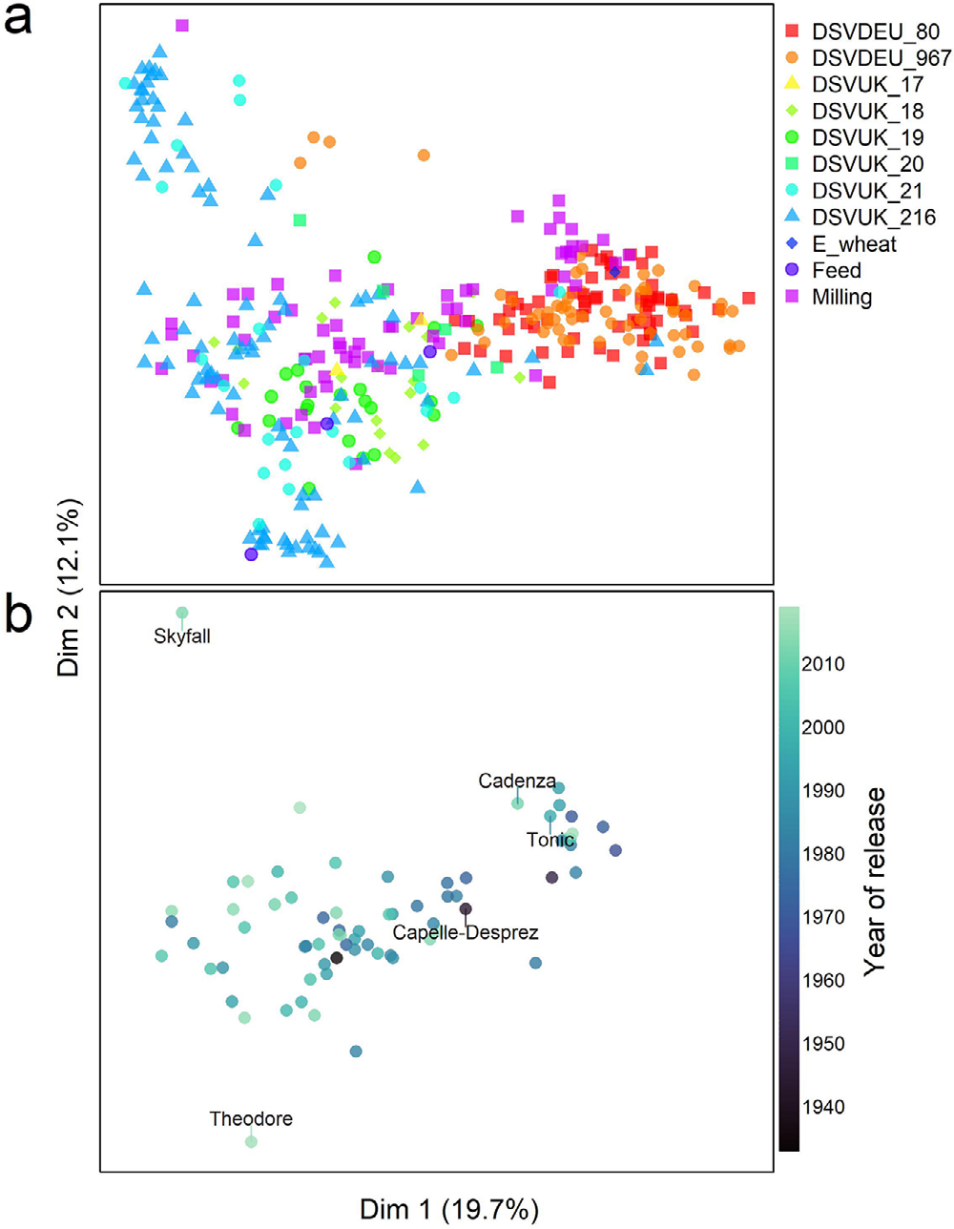
Principal coordinate analysis identifies population structure in the panel of wheat genotypes. The upper plot (a) shows differentiation of genotypes from the German and UK cycles of the Deutsche Saatveredelung (DSV) breeding program as well as released varieties. Details of genotypes in each group are shown in Table S1. DSVUK and DSVDEU groups indicate breeding lines form different cycles of the UK and German DSV breeding programs. E wheat varieties are a class of high‐quality and high protein content varieties in Germany. The lower plot (b) shows the distribution of released varieties according to year of release.

A panel of wheat varieties was assembled that represent major high‐quality milling wheat varieties released in the United Kingdom over recent decades and material within the DSV UK and Germany wheat breeding program. Genotypic relationships were characterized using data from a SNP genotyping array, and PCoA revealed clear structure in the panel (Figure 2). The first dimension, which explained 19.7% of the variation, largely differentiated between the German and UK DSV breeding lines (Figure 2a). The released milling wheat varieties were distributed evenly across the first PCoA dimension, but the older of these varieties, such as Cappelle Desprez, or more recent spring type varieties, such as Cadenza and Tonic, were more central between the UK and German groups, reflecting their ancestral significance across European breeding programs. The second PCoA dimension explained 12.1% of the variation and contrasted between clusters of milling and feed wheat quality DSV breeding lines that are more closely related to the modern but genetically distinct milling variety Skyfall and the modern feed wheat variety Theodore, respectively (Figure 2b). Differentiation between classes of German breeding lines was unclear within the first two PCoA dimensions, but a few lines were clustered toward the group of recently developed UK breeding lines, suggesting more recent exchange of material between countries.

**FIGURE 3:**
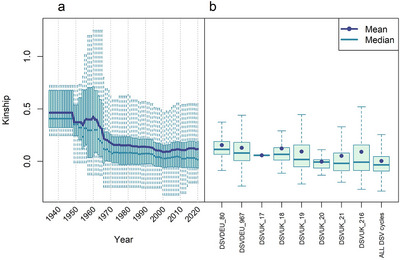
Trends in kinship indicate high and stable diversity among released milling wheat varieties over time (a), as well as within and among cycles of the UK and German DSV breeding program (b). Boxes indicate the upper and lower interquartile range and dashes indicate 1.5 × the inter quartile range.

A comparison of the relative kinship relationships among varieties released over time, and among breeding lines within each cycle of the DSV breeding program, revealed trends in genetic diversity (Figure 3a). It also allowed visualization of the level of diversity that breeders utilize within a breeding program (Figure 3b). The decrease in average kinship among released milling wheat varieties, particularly between 1950 and 1970, represents an incease in genetic diversity in that period, which has been stably maintained (Figure 3a). In comparison, the mean kinship among breeding lines within each of the UK and German cycles of the DSV breeding program are low (Figure 3b). Furthermore, mean kinship among lines across all UK and German breeding program cycles was even lower, suggesting greater genetic diversity is available to breeders where material is exchanged and crossed among countries and breeding cycles.

### 3.2 Phenotypic quality traits vary across years and breeding cycles

**FIGURE 4:**
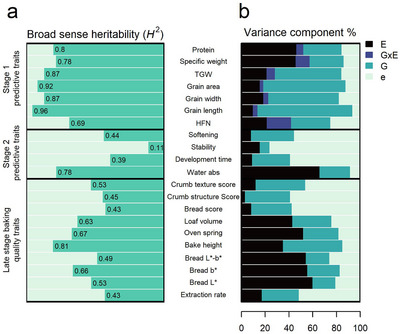
Comparison of trait heritability (a) and variance components (b) for grain and quality traits. E, environmental effects; G, genetic effects; G×E, genotype by environment interactions; e, residual error. G×E variance components could only be estimated for traits measured with replication within trials. HFN, Hagberg falling number; TGW, thousand grain weight; bread b*, bread yellowness reflectance; bread L*: bread whiteness reflectance.

The panel was characterized for a comprehensive set of predictive quality traits that are measured at early‐stages of the breeding program as well as more costly milling and loaf baking quality traits that are typically measured at later stages. These trials were also integrated with advanced breeding line trials as part of the DSV UK breeding program so that a meta‐analysis of predicted trait values across several years of unbalanced trials was possible and performed within a commercial breeding context. Estimation of trait heritability across these environments revealed that grain dimension traits (length, width, area, and thousand grain weight) had the highest heritability (*H* ^2^> 0.87) with only small environmental effects (Figure 4). Early‐stage predictive quality traits, including grain protein content, specific weight, and HFN, were generally also highly heritable (Figure 4a), but with larger environmental effects, and HFN had a relatively large genotype by environment interaction (G×E) variance component across the seven trial year environments (Figure 4b). Dough rheology stability had very low heritability (*H* ^2^ =0.11) due to a large residual error component, while the similar trait softening was greater (*H* ^2^ = 0.45). Among later stage milling and baking quality traits, loaf baking height and oven spring had the highest heritability. In contrast, the subjective loaf scores for overall baking quality showed only small environmental effects but had large error variance and therefore lower heritability. Bread color traits had much larger environmental effects but with only small error, and yellowness (bread b*) was more heritable than whiteness (bread L*) (Figure 4a).

### 3.3 Temporal trend analysis indicates quality trait changes over time

**FIGURE 5:**
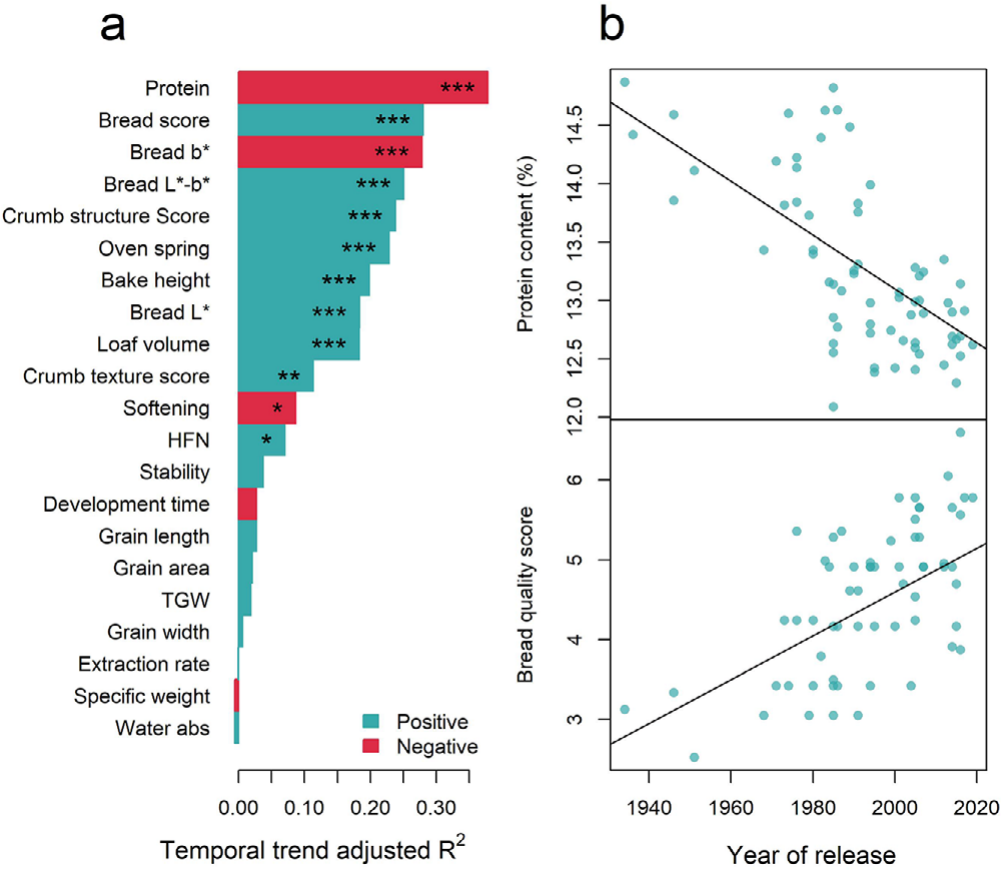
Temporal trends in wheat quality traits among released high‐quality UK wheat varieties. HFN, Hagberg falling number; TGW, thousand grain weight. For each trait, R2 is shown in (a), while (b) shows the linear regression for grain protein content and bread quality score against variety year of release. *** indicates *p* < 0.001, ** indicates *p* < 0.01 and * indicates *p* < 0.05.

Estimates of wheat quality traits were made for UK commercial milling quality varieties that were released over the last 85 years. Trends in these quality traits were quantified over this period of breeding by simple linear regression of the variety trait values against their year of release (Figure 5). The most prominent trend was a decrease in grain protein content despite its historical value as a trait for wheat bread quality. The data indicate that it has decreased by an average of 0.023% points per year so that a decrease of approximately 15% (from over 14.5% to just over 12.5% grain protein content) was observed over the period represented in this study (Figure 5). Despite this decrease in grain protein content, overall bread quality score has significantly increased (Figure 5b). This seems to be related to associated improvements and increases in loaf quality traits such as bread color (decreased bread b* and increased bread L*), larger loaf size (increased loaf volume, bake height, and oven spring), as well as better crumb texture and structure scores (Figure 5a). Other dough rheology traits including dough softening and stability also improved, but with a less clear trend. This generally suggests that the decrease in protein quantity has been compensated by increased gluten quality and has resulted in increased overall baking quality for the Chorley Wood baking process method tested here. HFN also showed a slight improvement, but other important milling quality traits such as flour water absorption, grain size, specific weight, and flour extraction rate show very little temporal trend.

### 3.4 Meta‐QTL analysis identifies known and novel genomic regions contributing to wheat quality traits

QTL mapping was performed by genome wide association for all traits in each of the two trial years and for trait values estimated across all environments by meta‐analysis. Across all traits and analyses, 337 marker trait associations were found, which could be grouped into 24 independent QTL based on linkage disequilibrium. Multi‐QTL models were fitted for each trait. Seven of the QTL were found to collocate for different traits (Table 1). QTL physical intervals and flanking markers were defined and gene content on the Chinese Spring RefSeq v1.0 genome was extracted. QTL intervals contained an average of 15 genes with a wide range of functions so identifying candidate causal genes was difficult (Table S2). The *QTgw.niab‐5A* QTL spanned a particularly large interval over 98 Mb across the centromeric region of chromosome 5A. Gene lists from identified QTL may be further reviewed for targeting potential as candidate genes.

**TABLE 1:** Analysis identified QTL for all traits across all environments (Meta) and within each main trial year (Yr1 and Yr2)

QTL	Trait	Chrom.	−log10(*p*)	Effect	*R* ^2^
*QHfn.niab‐1A*	HFN Meta	1A	4.00	19.258	0.04
*Glu‐1A*	Bread b* Yr2	1A	4.24	1.508	0.16
*Glu‐1A*	Bread L*‐b* Yr2	1A	4.91	−4.063	0.18
*QOsp.niab‐1A*	Oven spring Yr2	1A	5.48	−13.516	0.21
*QGl.niab‐1B*	Grain area Meta	1B	4.65	1.251	0.07
*QGl.niab‐1B*	Grain length Meta	1B	5.33	0.309	0.08
*QGl.niab‐1B*	Grain length Yr1	1B	4.91	0.310	0.08
*QGl.niab‐1B*	Grain length Yr2	1B	4.69	0.322	0.07
*QHfn.niab‐1D*	HFN Meta	1D	3.58	−23.379	0.04
*QSpw.niab‐1D*	Specific weight Yr1	1D	5.01	−1.032	0.08
*Glu‐1D*	Bake height Meta	1D	8.30	−7.825	0.15
*Glu‐1D*	Oven spring Meta	1D	7.34	−5.399	0.14
*Glu‐1D*	Bread score Meta	1D	6.88	−0.535	0.13
*Glu‐1D*	Crumb structure Score Meta	1D	6.31	−0.365	0.12
*Glu‐1D*	Bake height Yr1	1D	10.38	−15.870	0.33
*Glu‐1D*	Oven spring Yr1	1D	11.03	−15.131	0.35
*Glu‐1D*	Bread score Yr1	1D	9.44	−2.398	0.31
*Glu‐1D*	Crumb structure Score Yr1	1D	7.32	−1.632	0.24
*QGpc.niab‐2A*	Protein Yr2	2A	6.31	0.435	0.10
*QBcl.niab‐2B.1*	Bread b* Yr1	2B	5.50	0.674	0.18
*QBcl.niab‐2B.2*	Bread L* Yr1	2B	5.06	−3.202	0.17
*QBcl.niab‐2B.2*	Bread L*‐b* Yr1	2B	5.02	−3.960	0.17
*QBcl.niab‐2B.2*	Bread L Meta	2B	4.23	−0.789	0.08
*QBctx.niab‐2B*	Crumb texture score Yr2	2B	5.21	−2.481	0.20
*QBsc.niab‐2D*	Bread score Meta	2D	6.11	−0.535	0.11
*QBsc.niab‐2D*	Crumb texture score Meta	2D	7.87	−0.368	0.15
*QSpw.niab‐3A*	Specific weight Yr2	3A	5.22	1.245	0.08
*QBcl.niab‐3D*	Bread b* Yr1	3D	5.79	−1.166	0.19
*Phs‐A1*	HFN Meta	4A	3.52	15.447	0.04
*Phs‐A1*	HFN Yr1	4A	4.80	−49.083	0.07
*QTgw.niab‐5A*	TGW Meta	5A	6.12	2.344	0.09
*QTgw.niab‐5A*	Grain area Meta	5A	5.89	0.764	0.08
*QTgw.niab‐5A*	Grain length Meta	5A	5.97	0.178	0.09
*QTgw.niab‐5A*	TGW Yr1	5A	5.53	2.502	0.09
*QTgw.niab‐5A*	Grain area Yr1	5A	4.03	1.465	0.06
*QTgw.niab‐5A*	Grain area Yr2	5A	6.68	0.758	0.11
*QTgw.niab‐5A*	Length Yr2	5A	6.54	0.193	0.10
*Pinb‐D1*	Water abs Meta	5D	6.54	−1.668	0.14
*Pinb‐D1*	Water abs Yr2	5D	5.86	−2.822	0.22
*QSof.niab‐6B*	Softening Meta	6B	4.92	−7.299	0.10
*QHfn.niab‐6D*	HFN Yr2	6D	5.66	29.151	0.09
*QGrw.niab‐7A*	Grain width Meta	7A	5.24	−0.064	0.07
*QHfn.niab‐7B.1*	HFN Meta	7B	3.41	−23.252	0.03
*QHfn.niab‐7B.2*	Softening Yr1	7B	7.31	56.067	0.31
*QHfn.niab‐7B.2*	HFN Meta	7B	3.18	−28.770	0.03
*QExr.niab‐7D*	Extraction rate Yr1	7D	4.95	1.349	0.16
*QHfn.niab‐Un*	HFN Meta	Unmapped	3.33	−17.254	0.03

*Note*: *R* ^2^ and −log10(*p*) values were derived from multi‐QTL models for each trait.

Abbreviations: HFN, Hagberg falling number; QTL, quantitative trait loci; TGW, thousand grain weight.

Some of the QTL could be related to previously characterized QTL in the literature based on physical map position. The largest effect QTL found for several baking loaf size and bread quality traits measured in year 1 and in the meta‐analysis was mapped to a similar position as found by Fradgley et al. (2022) for dough rheological traits, and BLAST search for the gene sequence provided by Anderson et al. (1989) indicated that TraesCS1D02G317200 is the likely candidate gene in the interval. This QTL was, therefore, considered to distinguish between the positive effect 5+10 allele and other lower score alleles at the *Glu‐1D* locus (Table 1). Similarly, a QTL on chromosome 1A was found to have an effect on bread color reflectance traits, was close to the homoeologous region to *Glu‐1D* as also identified by (Fradgley et al., 2022), and was within 4 Mb of results from the BLAST search for gene sequence provided by De Bustos et al. (2000) and so was considered to probably be the *Glu‐1A* locus (Table 1). Although *Glu‐1A* would be expected to have an effect on gluten strength and loaf volume traits, the novel effect on bread color may be due to an associated measured color difference in enhanced bread crumb texture. The allele that significantly increased bread color yellowness (b*) in year 2, also decreased the crumb structure score from a mean of 3.8 to 2.6 and loaf volume by 8.2% in year 2, but these effects were not genome‐wide significant.

Of the seven QTL found for HFN, two could be related to genes or QTL previously found in the literature. First, the QTL on the long arm of chromosome 4A with a peak marker at 605,664,305 bp was found to match the pre‐harvest sprouting (*Phs‐A1*) locus because it had close synteny with the *Triticum dicocoides* 4A region between 595.3 and 596.7 Mb as described by Shorinola et al. (2017). BLAST search for the sequence of the *TaMKK3‐A* candidate gene provided by Torada et al. (2016) mapped to an unknown chromosome, but of the gene models in the defined interval, TraesCS4A02G315500 included “embryo development ending in seed dormancy” as a Gene ontology (GO) term. Second, the QTL on chromosome 7B (*QHfn.niab‐7B.2*) at 750,605,912 bp was found to approximately collocate with a QTL at 750,082,927 bp that explained a relatively large proportion of the phenotypic variance in HFN by (White et al., 2022) and is in a similar region to QTL identified by Mohler et al. (2014) and a near significant QTL identified in Danish breeding lines by Kristensen et al. (2018). However, neither of these QTL was found to explain a large proportion of the trait variation in HFN across multiple environments (*R* ^2^ < 0.04). Furthermore, the data showed that *QHfn.niab‐7B.2* also had a pleiotropic positive effect on dough softening. A single QTL was found for flour water absorption at the distal region of the short arm of chromosome 5D and is a likely colocation with the known *Ha* hardness locus where puroindoline protein genes are located (Morris, 2002) and matched a BLAST search from sequence from Ayala et al. (2016).

Some potentially novel QTL that could not be related to others in the literature were found for predictive quality traits. Grain size traits (grain length, width, area, and TGW) had high heritability (*H* ^2^> 0.87), and two QTL on the long arm of chromosome 1B (*QGl.niab‐1B*) and over a wide interval of the centromeric region of 5A (*QTgw.niab‐5A*) were consistently found in both main trial analyses and meta‐analysis. Despite relatively high heritability of grain protein content and specific weight (*H* ^2^= 0.80 and 0.78, respectively), only single QTL were identified for each of these traits on chromosomes 2A (*QGpc.niab‐2A*) and 3A (*QSpw.niab‐3A*), respectively, and these explained a small proportion of the phenotypic variation (*R* ^2^ < 0.1) in only one of the main trial environments. HFN was found to be of lower heritability (*H* ^2^ = 0.69), but several QTL were found that explained a small proportion of the trait variation (0.03 < *R* ^2^ < 0.09). A particularly large G×E variance component was found for HFN, and none of the HFN QTL were identified in both main trial years.

Several further potentially novel QTL were also identified for loaf baking quality traits. In particular, two QTL were identified on chromosome 2B (*QBcl.niab‐2B.1* and *QBcl.niab‐2B.2*), and one on 3D (*QBcl.niab‐3D*) for loaf color traits measured by color reflectance, all explaining large proportions of the trait variation in year 1 main trials (*R* ^2^ > 0.17). However, none of these were identified in year 2 or the meta‐analysis. A potentially novel QTL on chromosome 2D (*QBsc.niab‐2D*) for overall bread score and crumb texture score was identified for the all‐trials meta‐analysis. Despite relatively low heritability of flour extraction rate (*H* ^2^= 0.43), a QTL on chromosome 7D (*QExr.niab‐7D*) was found for this trait in year 1 trials only, explaining a reasonably high proportion of the phenotypic variation (*R* ^2^ = 0.16).

### 3.5 Changing allele frequencies provide evidence for historic selection of specific QTL

The temporal trends in allele frequency of beneficial peak marker alleles identified by the QTL analysis among the panel of released varieties were examined using a 20‐preceding years sliding window moving average. This considered examples of varieties in the historic pedigree where breeders had selected the variety from a cross between polymorphic parents. Considering groups of QTL based on their effect on different wheat quality traits, the observed trends and shifts in allele frequency largely reflected and explained many of the changes found for different quality traits. The positive allele for *QGpc.niab‐2A* was found to be common among varieties before 1970, such as Bersee and Highbury, but rapidly declined in frequency among varieties released since then (Figure S1a). All four examples of milling wheat varieties that were found to be selected from parents that were polymorphic for *QGpc.niab‐2A* (Malacca, Maris‐Widgeon, Caxton and Chilton) inherited the low grain protein content allele suggesting that breeders have been actively selecting against this QTL.

The compensatory increase in loaf baking quality traits despite decreased protein content was explained by the steady increase in frequency of positive alleles for four QTL found here affecting loaf baking quality and crumb color traits (Figure S1b,c). Only one of these QTL (*QOsp.niab‐1A*) had a sufficient number of examples of varieties with known polymorphic parents to test pedigree selection effects. This analysis found that six of the seven varieties selected from polymorphic parents (Spark, Solitaire, Wembley, Ketchum, Gallant, and Kingdom) inherited the positive *QOsp.niab‐1A* allele, while only Xi‐19 did not. This suggests that breeders were actively selecting positive genetic effects within families of segregating breeding lines. While the QTL for loaf quality traits are almost all fixed for the positive allele among the most recently released milling wheat varieties, they generally remain polymorphic among breeding lines within the DSV UK breeding program, which include both high milling quality and low‐quality feed wheat varieties (Figure S1b). QTL for loaf color traits (such as *QBcl.niab‐3D*), show an increasing trend but are still at lower frequency in released varieties and among DSV breeding lines.

In contrast, the overall frequencies of positive alleles for identified QTL for other traits, such as HFN, grain size traits, and specific weight, generally remained neutral (Figure S1a). Although the positive allele for *QSpw.niab‐1D* seemed to increase in frequency, *QSpw.niab‐3A* showed the opposite trend and this also reflects the lack of temporal trends in these traits (Figure 5a). Taken together, these results show evidence of selection in line with changes in wheat quality traits over time and support potential use of QTL effects identified from GWAS.

### 3.6 Genomic prediction accuracy increases with model optimization based on trait genetic architecture

**FIGURE 6:**
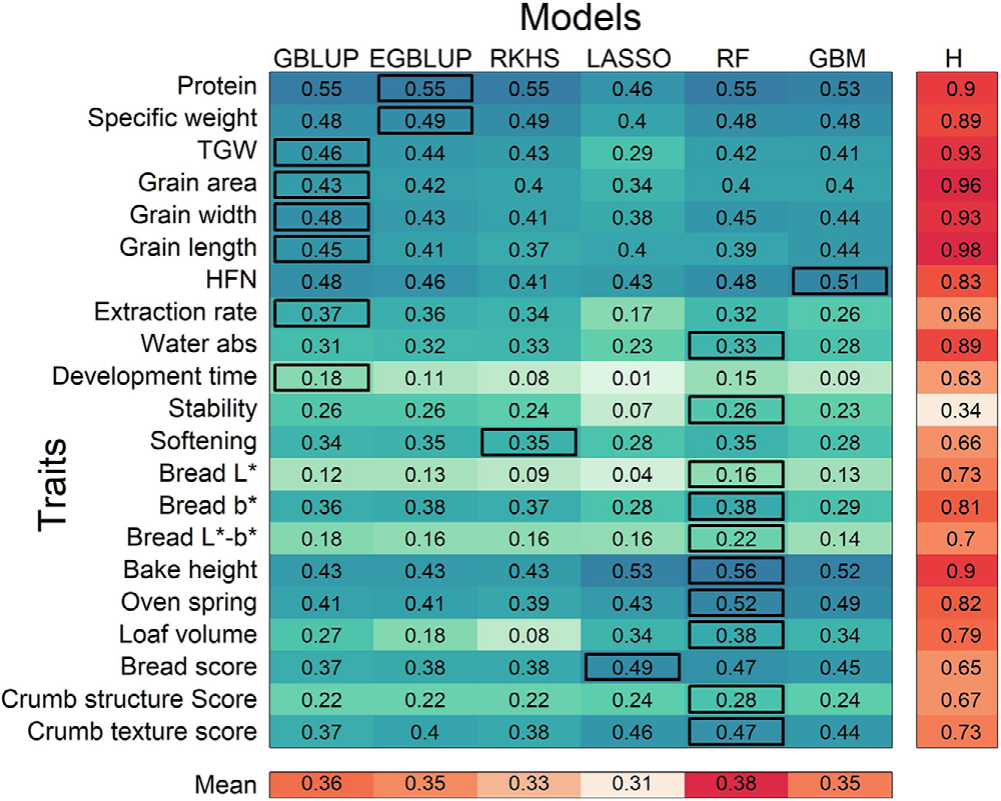
Model testing identified variation in mean prediction accuracy for all traits from random cross validation. Prediction accuracy is calculated as the Pearson correlation between the observed and predicted trait values. Black boxes indicate the most accurate model for each trait. The theoretical maximum prediction accuracy derived from the square root of the trait broad sense heritability (H), indicating the theoretical maximum prediction accuracy, is shown to the right. TGW, thousand grain weight; HFN, Hagberg falling number.

To determine the suitability of using genomic selection for wheat milling and baking quality traits, a diverse range of genomic prediction models whose assumptions encompass several different trait genetic architectures were tested. Under a random 10‐fold cross validation scheme, generally high prediction accuracies were achieved considering the theoretical maximum accuracy based on the estimated heritability of each trait (Figure 6). More than half of the heritable genetic variation could be predicted for traits such as grain protein content, specific weight, or HFN, as well as some of the loaf quality traits including overall bread score and baking height. For traits including flour water absorption and bread L*, a much smaller proportion of the heritable variation could be predicted. Overall, much greater potential to predict heritable trait variation could be demonstrated from genomic prediction than the combination of all identified QTL. This was particularly so for traits such as protein content or specific weight for which QTL were only found in single environments and explained less than 10% of the phenotypic variation, whereas cross validation of genomic prediction models showed that over half of the heritable phenotypic variation could be successfully predicted. This suggests that genomic prediction models can successfully integrate a much larger number of smaller effect QTL to predict highly polygenic traits.

There was no single prediction model that performed best for all traits (Figure 6). However, as expected, the differences in model prediction accuracy appear to reflect the underlying genetic architecture of each trait. For example, LASSO models performed poorly in comparison to models based on estimates of kinship (GBLUP, EG‐BLUP and RKHS) for traits such as grain protein content, specific weight, and TGW suggesting that these are highly polygenic traits with many genetic effects distributed across the genome. Despite relatively high heritability, only a few QTL of small effect size, with low *R* ^2^ values, were detected for these traits (Table 1). LASSO was the most accurate model for overall bread score and performed relatively well for other related traits such as loaf volume and crumb texture score. Together with the large *R* ^2^ values for QTL identified for these traits, this suggests that a larger fraction of the variation is explained by a few large‐effect QTLs.

There was rarely an advantage of the more complex models that aim to account for epistatic genetic effects (EG‐BLUP and RKHS) over the baseline GBLUP models that include linear genomic relationship matrices, suggesting a minimal role of epistasis for most traits in this dataset. However, the machine learning based models (RF and GBM) were able to flexibly integrate and predict non‐linear and non‐additive effects, and these performed competitively for the range of traits. RF was often the most accurate model (for 10 out of 21 traits) or comparable with the most accurate model, especially for the loaf quality traits such as overall bread score or bake height. RF and GBM models share similar model structure, but RF performed better than GBM for all traits except HFN when GBM was the overall most accurate model.

**FIGURE 7:**
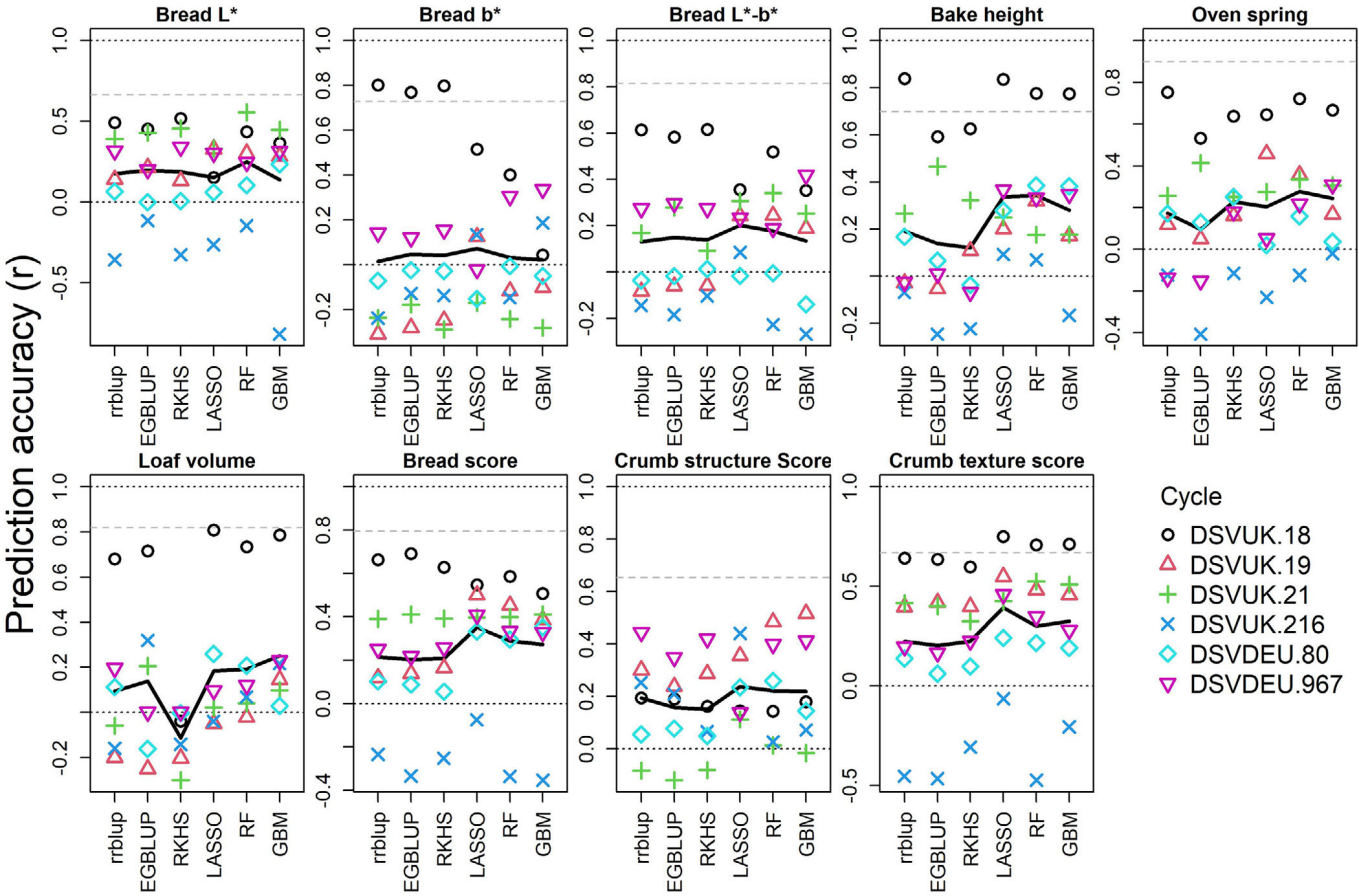
Prediction accuracy of different models for a subset of predictive and baking quality traits from cross validation for groups of lines from each DSV breeding program cycle. Pots for all traits are shown in Figure S2. Prediction accuracy was tested on lines from each cycle of the Deutsche Saatveredelung (DSV) UK and German breeding program (i.e., DSVUK.18) from models trained on all other data. Prediction accuracy is calculated as the Pearson correlation between the observed and predicted trait values. Solid black lines indicate the mean accuracy across all breeding cycles. Horizontal grey dashed lines indicate the theoretical maximum prediction accuracy derived from the square root of the trait heritability.

Practically, within a breeding program, genomic selection is used for forward prediction from past to future breeding cycles and genotypes become more distantly related to the training panel relative to random k‐fold cross‐validation across cohorts. Therefore, the accuracy from cross validation of genomic prediction models were also tested where lines from each cycle of the DSV UK and German breeding program were used as the test fraction and models were trained using all other data. Prediction accuracies were much more variable among breeding cycle cross validations than for multiple comparisons of random cross validation (Figures 6 and 7 and Figure S2). This was likely due to the smaller number of lines within some breeding cycles making accurate estimation of prediction accuracy difficult. While there was large variation in prediction accuracies between cycles, they were generally lower on average than for random cross validation for several traits. Traits such as grain protein content and HFN were predicted only slightly less accurately compared to random cross validation, whereas specific weight and grain size traits prediction accuracy was much lower for cross validation among breeding cycles.

Across all breeding cycles, trends among model accuracies generally reflected those from random cross validation. For example, LASSO models performed poorly for traits such as grain protein content, specific weight, and water absorption, but were more accurate for loaf baking quality traits such as overall bread score and crumb texture score. Similar to random cross validation, specific weight was better predicted using kinship‐based prediction models (GBLUP, EG‐BLUP, and RKHS), and HFN was best predicted using GBM. Trends in accuracy between different breeding cycles varied among traits. For example, lines from the DSVUK.18 breeding cycle were accurately predicted for most loaf baking quality traits, such as baking height and loaf volume, but poorly for grain size traits, such as TGW and grain length and width. Also, genotypes from UK and German breeding cycles were generally more genetically distinct (Figure 2), and prediction accuracy of both German cycles was lower for grain protein content when the models were trained on the remaining, mostly UK origin, genotypes. However, this trend was less evident for other traits.

### 3.7 Genomic versus phenotypic selection for high value, late‐stage quality traits

We considered the practical implementation of genomic prediction in a commercial context where early‐stage predictive quality traits are used to predict and make selections for later stage loaf baking quality traits. To do this, the prediction accuracy of genomic prediction models and phenotypic prediction models based on early‐stage predictive quality traits was compared. To be effectively used at earlier stages of the breeding cycle, genomic selection must, therefore, add significant predictive ability for the more costly later stage milling and loaf baking quality traits in addition to the predictive phenotypic data that are already available at this stage.

Considering correlations between individual predictive traits at the first stage (protein content, specific weight, HFN, and grain size traits) and second stage (additionally water absorption, development time, stability, and softening) of the breeding program, these traits were partially predictive of later stage loaf baking quality (Figure S3). Although correlations were generally low (*r* < 0.43), directions were as expected. Specific weight had the strongest correlation with flour extraction rate (*r* = 0.27), compared to grain size traits that were much lower (*r* < 0.09). Both grain protein content and grain width, rather than length, correlated positively with flour water absorption (*r* > 0.25). Specific weight also had clear correlations with other baking quality traits, particularly for bread whiteness L* (*r* = 0.26), while correlations with grain protein content were surprisingly low considering its importance as a basic measure of quality. Protein content was even negatively associated with some bread color traits where higher protein lines often had lower bread L* values (*r* = −0.13). The correlation between grain protein content and overall bread score was near zero, but this relationship was found to be non‐linear where the optimum protein content was estimated to be just over 13%, while either excessively high or low protein content lines generally had poorer bread score (Figure S4). Although there were many lines with protein content around 13% and very low overall bread score, the top 10% of lines with the highest bread score all had protein contents within a narrow range of 12.36% and 13.52%. Therefore, grain protein content is a poor predictor of baking quality alone, but must be around 13% for lines to be of high potential baking quality. HFN was also positively but weakly associated with many later stage baking quality traits (Figure S4).

Addition of second stage predictive traits for gluten quality showed that only dough stability and softening had strong associations with loaf baking quality traits (Figure S4). Softening had the strongest association with all loaf baking quality traits except bread b* and crumb color score suggesting good predictive potential for use in selection. Flour water absorption and development time had much weaker associations with baking quality traits. Overall, these results suggest that different predictive quality traits provide information relating to independent aspects of loaf baking quality so forward selection based on multiple traits would be required in a breeding program.

Considering the relationships among predictive quality traits and later stage milling and loaf baking quality traits, the predictive accuracy of multi‐trait phenotypic, single‐ and multi‐trait genomic, and trait‐assisted genomic prediction approaches were compared. Phenotypic prediction models were fitted using simple multiple regression with either sets of first or second stage predictive quality traits as explanatory variables. These were compared to single‐trait genomic selection using Bayesian mixed models as well as multi‐trait models for completely unphenotyped test lines. Furthermore, a trait‐assisted selection scenario was investigated where multi‐trait models were fitted and data for predictive quality traits were not masked from the test lines so that both genomic and trait data were used to predict test line phenotypes. Only the Bayesian mixed models with linear genomic relationship matrices were used here for comparisons between single and multi‐trait genomic prediction models.

**FIGURE 8:**
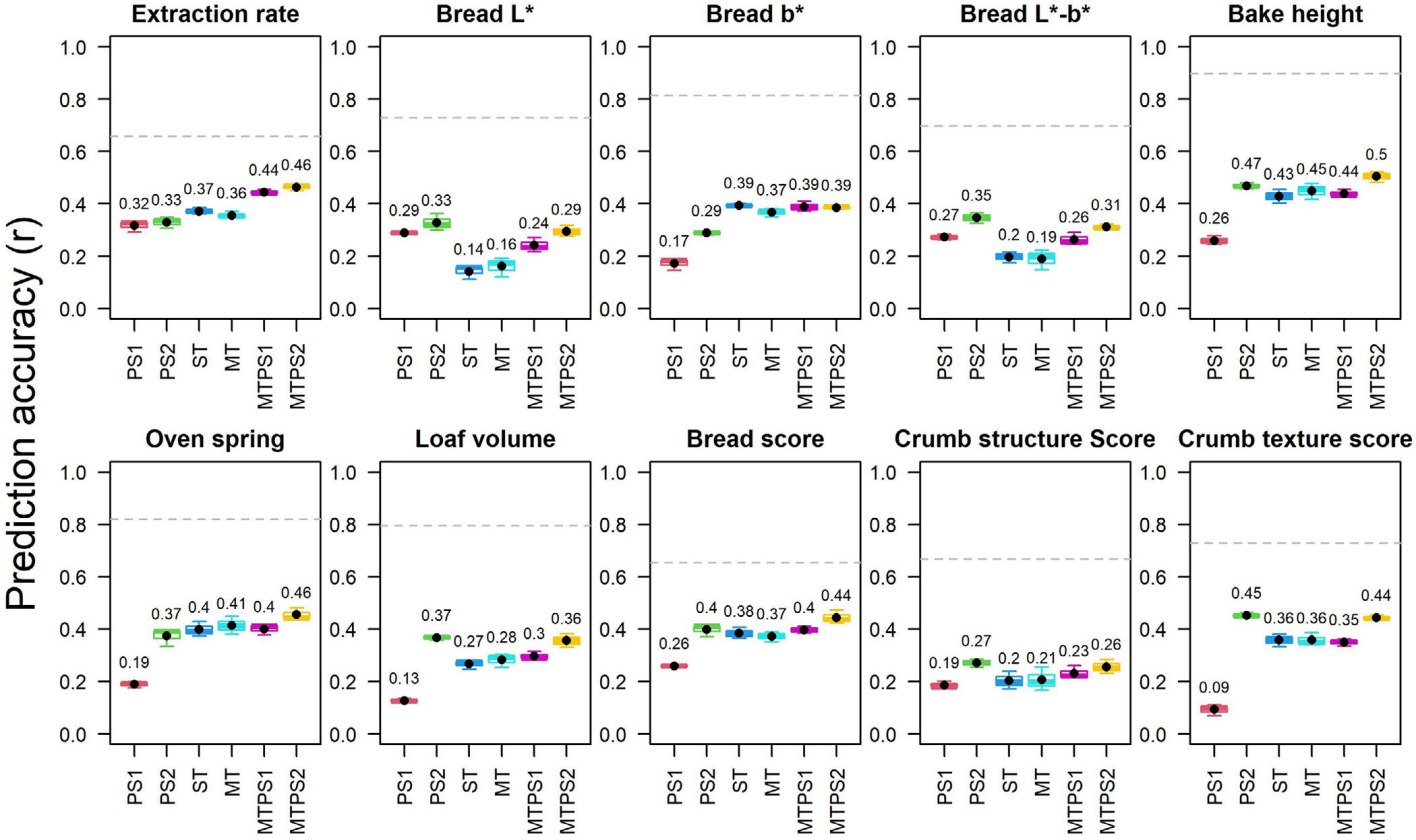
Prediction accuracy varies when comparing phenotypic as well as single‐ and multi‐trait genomic prediction models. PS1, phenotypic selection using stage 1 predictive traits (grain protein content, specific weight, and Hagberg falling number); PS2, phenotypic selection using stage 2 predictive traits (PS1 traits as well as DoughLab development time, stability, and softening); ST, single‐trait genomic selection; MT, multi‐trait genomic selection for completely unphenotyped test lines; MTPS1, multi‐trait‐assisted genomic selection with PS1 traits included for test lines; MTPS2, genomic selection with PS2 traits included as covariates. Boxplots show the distribution of prediction accuracies from each round of 10‐fold cross validation and black points indicate the mean. Horizontal dashed lines indicate the theoretical maximum prediction accuracy derived from the square root of the trait heritability.

Simple, multiple linear regression models were found to be the best approach for combining a small number of predictive traits but phenotypic selection with only the first stage predictive quality traits was generally inaccurate (Figure 8). Inclusion of the second stage dough rheology predictive traits increased accuracy of phenotypic selection considerably for all traits except flour extraction rate which was largely predicted by the strong association with grain specific weight. Genomic prediction using the single‐trait GBLUP model outperformed phenotypic selection with first stage predictive traits for all late stage milling and baking quality traits except for bread L* and bread L*−b* (Figure 8). Second stage phenotypic selection was more accurate than the single‐trait GBLUP for most loaf baking traits (Figure 8), but the best single‐trait genomic prediction models compared in Figure 6 were still generally more accurate than phenotypic selection for these traits, with the exception of bread L* and L*−b*.

These results suggest that similar accuracy of selection can be achieved from genomic selection as for phenotyping advanced second stage predictive quality traits. Multi‐trait genomic prediction models that consider covariance among traits did not significantly improve accuracy over single‐trait genomic prediction models for any trait when predicting completely unphenotyped lines. However, when using a trait‐assisted genomic prediction scenario where a breeder has genetic marker data for breeding lines that have already been phenotyped for either first or second stage predictive quality traits, multi‐trait models generally did have increased accuracy over single‐trait models. Similar to trends in phenotypic selection, the value of including first rather than second stage predictive traits in trait‐assisted genomic prediction models was greater for flour extraction rate, but for most loaf baking quality traits, there was little advantage in accuracy until second stage dough rheology traits are also measured on the test lines.

**FIGURE 9:**
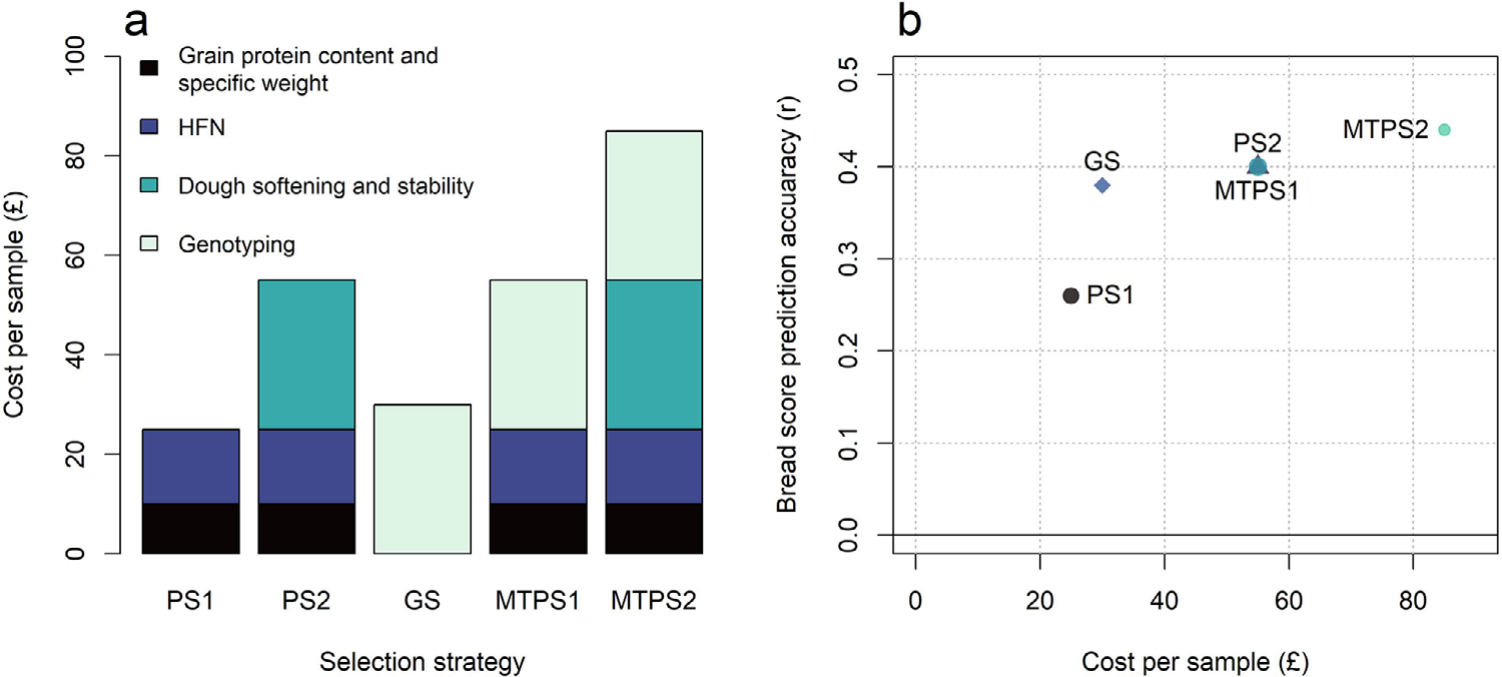
Cost per unit of accuracy varies between selection strategies: (a) breakdown of estimated costs among different phenotypic and genomic selection strategies and (b) comparison of selection strategy costs with the prediction accuracy of overall bread score. PS1, phenotypic selection using stage 1 predictive traits (grain protein content, specific weight, and Hagberg falling number); PS2, phenotypic selection using stage 2 predictive traits (PS1 traits as well as DoughLab development time, stability, and softening); GS, genomic selection; MTPS1, multi‐trait‐assisted genomic selection with PS1 traits included for test lines; MTPS2, genomic selection with PS2 traits included as covariates.

Although combining all stages of phenotyping with genomic prediction did increase prediction accuracy, this advantage may not be large enough to justify the costs of both phenotyping and genotyping. Considering the estimated genotyping and phenotyping costs involved in implementing phenotypic, genomic, and trait‐assisted genomic selection (Figure 9a), genomic selection offers the greatest value per cost prediction accuracy of overall bread score to enable efficient selection at only a slightly greater cost than for first stage predictive trait phenotyping (Figure 9b). Implementing trait‐assisted genomic selection where combined costs of both genotyping and phenotyping are much higher only offers a relatively small increase in accuracy. These results indicate strong potential for genomic prediction to be applied in a breeding program instead of costly second stage predictive quality trait phenotyping where the best suited single‐trait genomic prediction model for each trait can be applied with great effect.

## 4 DISCUSSION

Novel QTL identified here may be useful for early‐stage marker assisted selection within segregating families in breeding programs. While evaluation of wheat varieties’ suitability for baking quality has traditionally been based on several predictive quality tests, data show that direct genomic prediction of advanced baking quality traits holds great potential for accurate genomic selection compared to selection based on additive QTL detected through GWAS or phenotypic selection based on early‐stage predictive quality traits.

### 4.1 Trends in UK quality wheat breeding

Genetic analysis showed a decrease in mean kinship among released varieties, most notably until 1970, indicating that the diversity of wheat varieties released by breeders for quality has increased. There has been no subsequent net loss of genetic diversity due to breeders’ selection, despite significant genetic gain for both yield and quality traits being achieved during this time. This concurs with other studies that found little decline in diversity over recent decades of UK (Donini et al., 2000; Srinivasan et al., 2003; White et al., 2008) and German (Reif et al., 2005) wheat breeding. Concerted pre‐breeding efforts to introgress genetic diversity from the tertiary genepool (Leigh et al., 2022), as well as historic exchange of breeding material among countries and regions (Fradgley et al., 2019) are both likely to have countered loss of genetic diversity due to continued breeders’ selections and fixation of positive genetic effects controlling specific traits.

Results from analysis of kinship among wheat genotypes suggest that significant trends in genetic gain in quality traits have been achieved without net loss of genetic diversity. More modern milling wheat varieties in the United Kingdom generally have lower grain protein content compared to older varieties despite high grain protein being a desirable quality trait for selection. This aligns with other studies which found decreasing trends in protein content in line with the negative genetic trade‐off between yield and protein content (Scott et al., 2021; Shewry et al., 2016) and underlines the focus that breeding has had on increasing yield. However, the clear increase in loaf baking quality scores indicate that the reductions in protein content have been more than offset by increased gluten protein quality. Whereas other studies have found that grain protein content is well correlated with loaf volume (Groos et al., 2007; Ibba et al., 2020), the lack of a strong association presented here is likely due to the Chorley Wood baking process method used here. At the time of its development, this process reduced the protein quantity requirements of UK grown wheat (Cauvain & Young, 2006). We propose that selection for increased gluten quality in combination with changes in the industrial bread baking process have enabled the large increases in yield of wheat achieved through breeding in recent decades (Calderini & Slafer, 1998; Mackay et al., 2011). Similar trends in decreased protein content have been observed from studies in Germany (Laidig et al., 2017), Spain (Sanchez‐Garcia et al., 2015), and the United States (Fufa et al., 2005). However, analysis of the International Maize and Wheat Improvement Center (CIMMYT) wheat breeding program in Mexico, that focuses on a broader range of wheat end‐uses, suggest that increases in yield and functional quality have been possible without decreased protein content (Guzmán et al., 2017). CIMMYT has also taken the similar approach of specifically selecting for positive high‐ and low‐molecular‐weight glutenin gene alleles within crosses at early‐stages (Guzman et al., 2016). However, several other side‐effects of grain protein levels, both positive and negative, are important to consider in wheat breeding. Maintaining high grain protein content is important to prevent the dilution of wheat proteins such as albumins and globulins which are not functional for gluten strength but have valuable amino acid profiles (Lasztity, 2017), as well as associated mineral micronutrient density (Tabbit et al., 2017). Development of wheat varieties that have good loaf baking quality at low protein levels may also help reduce nitrogen fertilizer application requirements for milling wheat crops (Shewry et al., 2020), which is an increasingly important requirement for mitigating environmental costs and political uncertainties of food production (Bentley, 2022). Selecting for wheat varieties that optimize trade‐offs between yield, protein quantity, quality, and nutritional value will require a clearer understanding of the genetic control and prediction of specific wheat milling and baking quality traits.

### 4.2 Selection history of wheat quality QTL

Trends in QTL frequencies identified here for protein content and loaf quality traits reflect trends in these quality traits over time and suggest how historic progress has been made, as well as identifying loci which still have potential for future selection of positive alleles. The positive allele for the protein content QTL on chromosome 2A had been negatively selected and remains at low frequency in current breeding material so is likely involved in the trade‐off between yield and protein. It would therefore be a poor candidate for marker assisted selection. In contrast to the protein content QTL, the four QTL for loaf baking quality have been under strong positive selection and are all almost fixed in recent high quality milling wheat varieties but are more frequently polymorphic among the recent cycles of DSV UK breeding lines. While some of these QTL, such as *Glu‐1D*, are already well characterized and widely used in breeding programs, the novel QTL identified here, such as for oven spring and crumb texture, will be more easily applied in breeding, particularly for crosses between milling quality and higher yielding feed wheats, as practiced in the DSV breeding program.

Other important quality traits such as HFN, specific weight, and extraction rate were not found to have increased in more recent varieties as much as loaf baking quality traits, but some novel QTL for these traits were identified here. In particular, two QTL for HFN were likely co‐located with the *Phs‐A1* gene on chromosome 4A that has recently been fine mapped by Shorinola et al. (2017) as well as a QTL on 7A, which is likely concurrent with QTL identified by White et al. (2022) and Mohler et al. (2014). Although both of these QTL were only found in single trial years, there was a relatively large estimated G×E variance component of HFN. Therefore, use of multiple markers for environment specific QTL will be particularly important to select for increased stability of HFN in future years with unpredictable G×E effects rather than finding a QTL that has a consistent effect in all environments.

### 4.3 Genomic prediction offers improved selection over marker‐assisted and phenotypic selection based on predictive traits

QTL identified here will be useful for marker‐assisted selection using single markers to screen segregating families of breeding lines as early as the F2 generations to fix positive alleles. This works well for large effect QTL that explain a significant proportion of the trait variation. For complex traits such as grain protein content and specific weight, using multiple detected QTL can only account for a relatively small proportion of the trait variation and so making predictions of trait values is difficult. Genomic prediction models tested here made more accurate predictions explaining more than half of the heritable trait variation for most traits when using random cross validation. Cross validated prediction accuracy among cycles of breeding lines was lower but still demonstrated similar trends among prediction model performance and highlights the suitability of genomic selection in an applied breeding context.

Testing diverse genomic prediction models showed that no single model was the most accurate for all traits, thus highlighting the importance of testing multiple approaches for realistic breeding prediction scenarios and traits with unknown genetic architecture. In general, prediction accuracies were as expected for traits such as grain protein content and thousand grain weight, but lower for some traits such as specific weight, in comparison to other studies (Battenfield et al., 2016; Sandhu et al., 2021, 2022), but more similarly to Lado et al. (2018) which may be due to the lower number of genotypes in the panel analyzed here. Heritability of dough rheology traits including softening and stability was lower than expected in comparison to a previous study using the same method (Fradgley et al., 2022), and prediction accuracy was lower than for similar alveograph traits used in other studies (Kristensen et al., 2019; Lado et al., 2018). Similarly, prediction accuracy of loaf baking quality traits were high in comparison to some earlier stage predictive traits but were still lower than for studies with large breeding program datasets (Battenfield et al., 2016; Ibba et al., 2020), but were similar to Hayes et al. (2017) and higher than results from Longin et al. (2020) who used a much smaller panel.

In terms of differences among models, results presented here were in contrast to other studies where semi‐parametric models such as EG‐BLUP that include epistatic effects (Jiang & Reif, 2015; Martini et al., 2017) or modeling kinship using a Gaussian kernel with RKHS (de los Campos et al., 2010; Gianola & van Kaam, 2008) generally increase prediction accuracy over standard GBLUP. The poor performance of EG‐BLUP and RKHS models presented here may be down to how optimum values of the bandwidth parameter must be determined or the strong kinship structure in the panel. LASSO models performed most unreliably of all models, reflecting the polygenic genetic architecture of most traits investigated here. LASSO models work particularly well for relatively simple traits with only a few and additive genetic effects (Scott et al., 2021), so the good performance of these models for most loaf baking quality traits supports the evidence from the multiple major QTL effects such as the *Glu‐1D* locus that explained a high proportion of variation in these traits in GWAS. The non‐parametric machine learning models including random forest and gradient boosting machines seemed to perform competitively for most traits demonstrating their flexibility to model a broad range of genetic architectures. This is likely due to their ability to take complex non‐additive genetic effects into account (Niel et al., 2015). Charmet et al. (2020) also found that random forest had the highest accuracy for predicting wheat yield compared to a wide range of other models. Despite the complex and comprehensive hyperparameter tuning method used here, which requires extended computational time, gradient boosting machine models performed slightly worse than random forest for all traits except HFN. However, other studies have found that GBM models are able to offer better accuracy over random forest (Ogutu et al., 2011), and Montesinos‐López et al. (2022) found that GBM outperformed a Bayesian threshold GBLUP model in multiple datasets. These results demonstrate good prospects for more generally applying machine learning approaches to genomic prediction, and other deep learning approaches are likely to become increasingly useful for larger datasets (Montesinos‐López et al., 2021).

Multi‐trait prediction models which model the covariance structure among response variable traits were also tested. For cross validation scenarios where no phenotypic data are available for test lines, no increase in accuracy was found compared to single‐trait models. This is in contrast to findings for predicting some similar quality traits by Sandhu et al. (2022). However, cross validation schemes which included some predictive trait data for test lines did often show improved prediction accuracy over both phenotypic selection and single‐trait genomic prediction models. This demonstrates potential to maximize prediction accuracy by combining both trait‐assisted genomic selection programs. The benefit of multi‐trait models only when correlated traits are measured on test genotypes aligns with several others taking this approach, such as Zhang‐Biehn et al. (2021) who found similar results for predicting wheat baking quality traits and Lado et al. (2018) who used predictive quality traits to aid prediction of dough alveograph traits. In theory, a multi‐trait approach should take advantage of correlations among related traits with phenotypes only in the training set for simulated datasets (Calus & Veerkamp, 2011; Jia & Jannink, 2012). However, even other studies making multi‐trait predictions of yield do not find this in real data (Sun et al., 2017). The advantage of these models are likely to be greatest when secondary traits are highly correlated and have greater heritability than the target trait (Montesinos‐López et al., 2021, 2022), but this is rarely the case in breeding datasets. Here, we found that the dough rheology traits including stability and softening had surprisingly low heritability compared to other studies (Battenfield et al., 2016; Lado et al., 2018). This seemed to be due to a large error component, particularly in the second year of main trials, and may explain the lack of advantage of multi‐trait genomic prediction models.

The combined costs of both genotyping for genomic selection and phenotyping may not outweigh the benefit of increased accuracy from trait‐assisted genomic selection. Therefore, multi‐trait prediction of completely unphenotyped breeding lines that would be produced from speed breeding and genomic selection programs remains a challenge but of high relevance when using genomic predictions in multi‐trait selection indices (Moeinizade et al., 2020). Trait‐assisted genomic selection also likely achieves an increase in accuracy by the secondary traits accounting for G×E in the test material and models that integrate covariances between traits, genotypes, and environments have been developed (Montesinos‐López et al., 2016). Therefore, the enhanced prediction accuracy of trait‐assisted genomic prediction models found here within the same set of environments is likely overestimated compared to making predictions into separate unknown environments. Furthermore, the value of genomic prediction models that are trained on several years of previous trials will be greater for achieving long‐term genetic gain in future years with unpredictable G×E effects compared to phenotypic selection that can only be made in a limited number of years.

### 4.4 Best use of genomic selection in an applied wheat breeding program

In the context of an applied breeding program, a few generations of inbreeding to generate recombinant inbred lines (typically F3 of F4) are required before genotyping for genomic selection. Genomic prediction must therefore be competitive in terms of cost and accuracy compared to phenotypic selection based on predictive traits that can be measured on large numbers of breeding lines at the same stage. In the DSV UK breeding program compared here, the first stage predictive traits: protein content, specific weight, and HFN are tested on F4 breeding lines while second stage predictive traits dough stability, softening, and development time are tested using a DoughLab at F5 generations and are used to select a much smaller subset of lines to go forward for expensive loaf baking tests for late‐stage selection. Using first‐stage predictive traits in multiple regression models made generally inaccurate predictions of loaf baking quality traits but relatively good predictions of flour extraction rate and bread crumb whiteness (L*) due to the high correlation between these traits and grain specific weight. Groos et al. (2007) also showed the difficulty in predicting baking performance from predictive quality traits in a French study but found a stronger positive association between protein content and bread score which may be due to the differences in bread making methods used. Here, phenotypic selection was only competitive with the best genomic selection models for loaf baking quality traits once second stage dough rheology traits were measured, and these are much lower throughput and costly to run. However, other higher throughput measures of gluten quality, such as Zeleny and SDS sedimentation values (Preston et al., 1982; Zeleny et al., 1960) or solvent retention capacity (Kweon et al., 2011), may offer an effective intermediate phenotyping stage prior to the stage 2 dough rheology testing presented here. Hayes et al. (2017) also presented a novel method for integrating near infrared (NIR) and nuclear magnetic resonance (NMR) measurements on only small samples of flour in genomic prediction models. Nevertheless, comparison of results between phenotypic and genomic prediction demonstrated that effective selections for bread baking quality can be made using genomic predictions at earlier stages of the breeding program and at lower costs than early‐stage phenotyping, while predicting late‐stage baking traits with similar accuracy to the predictions from stage 2 phenotypic data.

As well as improving and accelerating forward selection of breeding lines with good expected loaf baking quality, genomic selection without the need for slow early‐stage phenotyping from field grown material enables much faster early generation times to develop sufficiently inbred line material by making use of recently advanced methods in speed breeding technologies (Cha et al., 2022; Ghosh et al., 2018). In this way, breeding lines with high genomic estimated breeding value can be recycled for crossing at earlier stages to minimize the breeding cycle time and maximize genetic gain in applied breeding programs (Alahmad et al., 2022; Voss‐Fels et al., 2019). Once a sufficiently large genomic prediction training dataset is established, genomic selections can be made on large numbers of completely unphenotyped breeding lines, and the training dataset can be kept up to date and accurate by incorporating phenotypic data from previous cycles of advanced line evaluation trials. In practice, genomic selection is of greater value as other important traits such as yield and disease resistance can also be included and predicted at the same cost of genotyping and selection indices can be applied to optimize genetic gain in relation to economic value of multiple traits (Céron‐Rojas & Crossa, 2018).

## 5 CONCLUSIONS

We report trends in wheat quality traits over the last half a century of intensive wheat breeding in the United Kingdom. Continuous selection for decreased protein content has been compensated for by increased gluten quality to achieve improved bread baking quality using current industrial baking methods. Identified QTL for quality traits changed in frequency over time, revealing the genetic basis for trait variation over time as well as future opportunities for marker‐assisted selection and improvement of quality traits that have been historically overlooked. We also demonstrate that genomic selection and trait‐assisted genomic selection hold great potential for cost‐effective selection and improvement of otherwise expensive milling and loaf baking quality traits in a commercial wheat breeding program context.

## AUTHOR CONTRIBUTIONS

**Nick Fradgley**: conceptualization; data curation; formal analysis; investigation; methodology; validation; visualization; writing—original draft; writing—review and editing. **Alison Bentley**: funding acquisition; investigation; methodology; project administration; supervision; validation; writing—review and editing. **Keith Gardner**: conceptualization; formal analysis; methodology; project administration; supervision; validation; writing—review and editing. **Stéphanie Swarbreck**: methodology; project administration; supervision; validation; writing—review and editing. **Matt Kerton**: data curation; funding acquisition; investigation; methodology; project administration; resources; supervision; validation; writing—review and editing.

## CONFLICT OF INTEREST STATEMENT

The authors declare no conflicts of interest.

## Supporting information


**Supplementary Figure S1**. Trends in frequency of positive effect alleles for identified QTL among released high quality UK wheat varieties and in different breeding cycles of advanced lines in the DSV UK breeding program. HFN: Hagberg falling number. For QTL details refer to Table 1.

**Supplementary Figure S2**. Prediction accuracy of different models for all predictive and late‐stage baking quality traits from cross validation for groups of lines from each DSV breeding program cycle. Prediction accuracy was tested on lines from each cycle of the DSV UK and German breeding program (i.e., DSVUK.18) from models trained on all other data. Prediction accuracy is calculated as the Pearson correlation between the observed and predicted trait values. Solid black lines indicate the mean accuracy across all breeding cycles. Horizontal grey dashed lines indicate the theoretical maximum prediction accuracy derived from the square root of the trait heritability.

**Supplementary Figure S3**. Pearson correlation coefficients between early‐stage predictive traits and later stage milling and baking quality traits. Coefficients in larger and darker font are statistically significant (*p* < 0.05).

**Supplementary Figure S4**. The non‐linear relationship between grain protein content and overall bread score BLUPs from trials analysis across all trial environments for all genotypes in the panel. The solid red line represents a spline fit with smoothing parameter: 1.2. The vertical dashed red line indicates the grain protein content for which fitted values of overall bread score is maximum.

**Supplementary Table S1**. List of historic and recently released wheat varieties included in the panel. Selection group indicates the reason for inclusion in the panel as outlines in the text above.

**Supplementary Table S2**. Lists of QTL intervals, gene content and gene GO terms.

## Data Availability

The genotypic and phenotypic datasets generated and analyzed in the current study are available at https://figshare.com/projects/Wheat_milling_and_baking_quality_dataset/153093. However, genetic marker names and genotype id names have both been anonymized due to commercial sensitivity of data relevant to the DSV UK breeding program.
